# Clinical and Psychological Factors Associated with Frailty in Patients with Heart Failure

**DOI:** 10.3390/jcm13237345

**Published:** 2024-12-02

**Authors:** Bernadetta Żółkowska, Christopher S. Lee, Quin E. Denfeld, Maria Jędrzejczyk, Dorota Diakowska, Magdalena Lisiak, Marta Wleklik, Michał Czapla, Izabella Uchmanowicz

**Affiliations:** 1 Student Research Club of Heart Diseases, Faculty of Medicine, Wroclaw Medical University, 51-618 Wroclaw, Poland; bernadetta.nowak@student.umw.edu.pl; 2 Boston College William F. Connell School of Nursing, Chestnut Hill, MA 02467, USA; leeddo@bc.edu; 3 School of Nursing, Oregon Health & Science University, Portland, OR 97239, USA; denfeldq@ohsu.edu; 4 Department of Nursing, Faculty of Nursing and Midwifery, Wroclaw Medical University, 51-618 Wroclaw, Poland; maria.jedrzejczyk@student.umw.edu.pl (M.J.); magdalena.lisiak@umw.edu.pl (M.L.); marta.wleklik@umw.edu.pl (M.W.); izabella.uchmanowicz@umw.edu.pl (I.U.); 5 Department of Midwifery, Faculty of Nursing and Midwifery, Wroclaw Medical University, 51-618 Wroclaw, Poland; dorota.diakowska@umw.edu.pl; 6 Institute of Heart Diseases, University Hospital, 50-556 Wroclaw, Poland; 7 Department of Emergency Medical Service, Faculty of Nursing and Midwifery, Wroclaw Medical University, 51-618 Wroclaw, Poland; 8 Group of Research in Care (GRUPAC), Faculty of Health Science, University of La Rioja, 26006 Logroño, Spain; 9 Centre for Cardiovascular Health, Edinburgh Napier University, Sighthill Campus, Edinburgh EH11 4DN, UK

**Keywords:** heart failure, frailty, cognitive impairment, depression, elderly patients, hospitalization

## Abstract

**Background/Objectives**: Heart failure (HF) is a significant public health issue with high morbidity and mortality rates. This study aims to investigate the interrelationships between frailty, cognitive impairment, and depression in older adults with HF, specifically focusing on how the physical and neuropsychiatric dimensions of frailty contribute to cognitive decline. **Methods**: This study included 250 patients aged 60 years or older, diagnosed with HF and hospitalized for acute decompensated HF. The patients were assessed using standardized protocols for frailty, cognitive function, and depression. The frailty was evaluated using Fried’s phenotype criteria, cognitive function with MMSE and MoCA, and depression and anxiety with HADS and PHQ-9. Statistical analyses included univariable and multivariable linear regression to identify the predictors of frailty. **Results**: Of the 250 patients, 151 (60.4%) were identified as frail. The frail patients were older (mean age 73.58 ± 6.80 years) compared to the non-frail patients (mean age 70.39 ± 6.16 years, *p* = 0.0002). Significant differences were observed in the NYHA class, length of the hospital stay, and prevalence of diabetes mellitus. The frail patients had worse cognitive (MMSE: 27.39 ± 2.12 vs. 28.13 ± 1.72, *p* = 0.004; MoCA: 24.68 ± 3.65 vs. 25.64 ± 3.98, *p* = 0.050) and psychological outcomes (higher prevalence of marked depression based on HADS categories: 8.61% vs. 1.01%, *p* = 0.021; and PHQ-9 categories: severe depression: 2.65% vs. 1.01%, *p* < 0.001). **Conclusions**: Age, C-reactive protein (CRP) levels, and anxiety were identified as independent predictors of frailty in the patients with heart failure. Depression, cognitive dysfunction, and the length of the hospital stay showed significant differences between the frail and non-frail patients in the group comparisons but were not independent predictors.

## 1. Introduction

Heart failure (HF) represents a major public health issue with substantial morbidity, mortality, and economic burden globally. Despite advances in treatment and management strategies, HF continues to have a high prevalence, affecting an estimated 64 million individuals worldwide [1].

Cognitive impairments are commonly seen in patients diagnosed with HF, leading to higher rates of mortality, frequent rehospitalizations, and a diminished quality of life [2]. HF predominantly impacts memory and executive functions, affecting critical domains that significantly influence a patient’s capacity to manage basic self-care behaviors [3].Both systolic and diastolic HF are linked to decreased cognitive abilities, which can manifest as delirium in hospitalized patients or as mild cognitive impairment in more stable individuals [2].

Frailty has emerged as a critical issue in the management of HF, particularly in the advanced stages of this cardiac disease, where symptoms persist despite comprehensive medical and device treatments. The prevalence of frailty in patients with HF is notably high, with estimates suggesting that up to 50% of these patients exhibit signs of frailty [4]. However, these figures vary significantly across different studies, influenced by factors such as the population being studied, and the methods used to assess frailty. Frailty in patients with HF is closely linked to poorer outcomes, including an increased risk of hospitalization and mortality—about 50% higher than in their non-frail counterparts [5]. Research consistently shows that frail patients with HF experience more severe symptoms, such as dyspnea, and a decline in their quality of life [4].

Mood disorders, such as depression, are recognized both as a risk factor for and a consequence of having frailty [6,7]. Depression notably impacts cognitive functioning and is associated with both frailty and cognitive impairment in populations with cardiovascular issues. This suggests that mood disturbances may play a significant role in the relationship between frailty and cognitive decline [8,9]. Both depression and anxiety are prevalent in individuals with heart disease and HF, and they often co-occur with frailty syndrome [10,11]. Cognitive deficits in depression span various functions, including attention, executive functions, memory, and processing speed [12,13]. These impairments often persist even when depressive symptoms are in remission, with cognitive difficulties reported to be present in 85–94% of depressive episodes and 39–44% of them during remission periods [14]. In some instances of Major Depressive Disorder (MDD), higher cognition impairments may dominate the clinical picture, significantly impacting the patient’s life and leading to a condition often referred to as “pseudodementia”. Distinguishing early on between cognitive impairment due to neurodegenerative or vascular causes, which is likely to progress, and depression-related cognitive impairment, which may be reversible, presents a clinical challenge [15,16].

The primary objective of this study was to investigate the interrelationships between frailty, cognitive impairment, and depression in older adults with HF. This research aimed to examine how both the physical and neuropsychiatric dimensions of frailty, including depressive symptoms, would contribute to cognitive decline. We seek to establish whether frailty, compounded by depressive states, can act as a predictive marker for the development of cognitive impairments in this population.

## 2. Materials and Methods

### 2.1. Patient Population and Procedure

This study included 250 patients diagnosed with HF. The patients were enrolled in the Institute of Heart Diseases at the University Hospital in Wroclaw, Poland, between August 2022 and June 2023. Data collection was conducted during hospitalization after the treatment of acute decompensated HF (ADHF) and once clinical stability was achieved, but before hospital discharge. Trained assessors used a standardized protocol to collect the data from the patients. The inclusion criteria for participant enrollment in this study were as follows: age ≥ 60 years old, diagnosis of HF in accordance with the European Society of Cardiology (ESC) guidelines of 2016, duration of HF ≥ 6 months, New York Heart Association (NYHA) functional class II–IV, recent hospitalization due to acute HF, and intact cognitive function. Exclusion criteria included NYHA class I, a score of MMSE ≤ 24 points, identified and treated depressive disorders, and a lack of agreement to participate in the study.

### 2.2. Measurements

#### 2.2.1. Frailty

The frailty phenotype was evaluated according to the criteria established by Fried, encompassing five domains: slowness, weakness, weight loss, exhaustion, and low physical activity [17].

1. Slowness: measured by walking a distance of 4.6 m. A point is given if this distance is completed in ≥6 s for women > 159 cm tall or men > 173 cm;
2. Weakness: Assessed using an electronic hand dynamometer (Deyard, China). Handgrip strength was measured by averaging three separate measurements;
3. Unintentional weight loss: defined as an unintentional loss of more than 4.5 kg or 5% of body weight in a year;
4. Exhaustion: Evaluated using the Center for Epidemiological Studies Depression Scale (CES-D) questionnaire. The criterion is met if the patient answers “3–4 days” or “5–7 days” to question 7 (everything I did was difficult) or question 20 (I couldn’t get going) in the past week;
5. Low physical activity: Quantified using the Minnesota Leisure Time Activities Questionnaire, which assessed the type, frequency, and duration of the physical activities undertaken in the preceding two weeks. Reduced levels of physical activity were defined as an energy expenditure of less than 383 kcal for men and less than 270 kcal for women in the week preceding the survey.

The participants were classified as frail if they exhibited at least three of these characteristics [17].

#### 2.2.2. Cognitive Impairment

Two tests were used to measure dementia: the Mini-Mental State Examination (MMSE) and the Montreal Cognitive Assessment (MoCA). The Mini-Mental State Examination (MMSE) is widely recognized as a tool for cognitive assessment. The Polish version of the MMSE was employed to evaluate the cognitive function in patients, yielding a maximum score of 30 points. Scores below 24 suggest potential dementia [18,19]. The Polish version of the Montreal Cognitive Assessment (MoCA) was used [20]. This tool can differentiate between normal cognitive function, mild cognitive impairment (MCI), and early onset dementia. The MoCA has a maximum score of 30 points. In individuals without functional impairment, a score below 26 points suggests MCI. An additional point is awarded to those with less than 12 years of formal education [20,21].

#### 2.2.3. Depression and Anxiety

Two tests were used to measure depression and anxiety: the Hospital Anxiety and Depression Scale (HADS) and the Patient Health Questionnaire-9 (PHQ-9).

The Hospital Anxiety and Depression Scale (HADS) was specifically devised to evaluate the severity of anxiety and depression symptoms in patients with physical health problems, particularly in a hospital setting. The Polish version of this tool was used in the study [22]. The HADS is a self-administered rating scale that consists of 14 items, each scored on a 4-point Likert scale (ranging from 0 to 3). This instrument is specifically designed to measure levels of anxiety and depression, with 7 items dedicated to each subscale. The overall score is the cumulative sum of all 14 items, resulting in scores that range from 0 to 21 [23]. The psychometric properties of the Hospital Anxiety and Depression Scale (HADS) used in this study demonstrated acceptable internal consistency, with Cronbach’s alpha values of 0.75 for the Anxiety scale and 0.77 for the Depression scale [24].

The Polish version of the Patient Health Questionnaire-9 (PHQ-9) was used in the study. This nine-item questionnaire was engineered to serve as a screening tool for depression, particularly within primary care and various medical settings [25]. It includes 9 basic questions and 1 additional question, making it a comprehensive tool for assessing depression. The Polish version of the Patient Health Questionnaire-9 (PHQ-9) demonstrated a Cronbach’s alpha of 0.7, indicating acceptable internal consistency [25].

### 2.3. Statistical Analysis

The quantitative variables were estimated as a mean ± standard deviation (SD) or median and interquartile range (IQR) [Q1–Q3], depending on their distribution. The qualitative variables were described as numbers and percentages. Comparisons between the groups were performed using a Student’s *t*-test or a Mann–Whitney U test, as appropriate. A univariable and multivariable logistic regression analysis was performed to evaluate the association of frailty with the selected parameters. The variables with a *p*-value greater than 0.100 in the univariable analyses were excluded from the multivariable model. A Classification and Regression Tree (CART) analysis was conducted to identify the cut-off point for the age parameter for the performance of logistic regression analysis. All the statistical tests were two-sided, and *p*-values less than 0.05 were considered statistically significant. Statistical analyses were performed using Statistica software version 13.3 (Tibco Inc., Palo Alto, CA, USA).

## 3. Results

This study enrolled 250 patients with HF, of which 151 (60.40%) presented with frailty syndrome, while 99 (39.60%) did not exhibit signs of frailty. The patients with frailty were significantly older, with an average age of 73.58 ± 6.80 years compared to 70.39 ± 6.16 years for those without frailty (*p* = 0.0002). No significant differences were observed in gender, marital status, residence, work status, education years, BMI, central obesity, alcohol consumption, and smoking status between the two groups. (Table 1).

Significant differences were noted in the clinical assessments and comorbidities, particularly with NYHA class IV being more prevalent in the frail group. The patients with frailty had a longer hospital stay (*p* = 0.003) but a lower prevalence of diabetes mellitus (*p* = 0.032) (Table 2). The patients in the frailty group had significantly lower levels of hemoglobin and eGFR, and a significantly higher concentration of CRP and NT-proBNP than those in the non-frailty group (Table 2).

Statistical analyses highlighted that the frail patients exhibited significantly worse outcomes in the cognitive and psychological assessments, including higher levels of anxiety and depression, as well as lower MMSE and MoCA scores (Table 3).

Age, NYHA class, length of hospital stay, diabetes mellitus, a low concentration of hemoglobin and eGFR, a high level of CRP, and anxiety and depression scores were identified as significant predictors of frailty in the patients with HF (Table 1, Table 2 and Table 3). These parameters were included in univariable and multivariable logistic regression analyses. The NT-proBNP parameter was excluded from the analysis because it did not meet the assumptions of the regression analysis. The results obtained by univariable logistic regression showed that all the study parameters, except MMSE, were significant predictors of frailty. A multivariable regression analysis demonstrated that older age (*p* = 0.001), the presence of inflammation (high concentration of CRP) (*p* = 0.045), the presence of anxiety (*p* = 0.008), and depression (according PHQ-9 scale, *p* = 0.004) were associated with frailty syndrome among the HF patients (Table 4).

## 4. Discussion

Several significant factors were identified, highlighting the multifaceted nature of frailty in the population of frail patients with HF. Firstly, age, as a non-modifiable factor, unsurprisingly played a critical role in the development of frailty as indicated in this study. It turned out that older patients with HF had more pronounced signs of frailty, which can be attributed to the cumulative decline in physiological reserves and the increased vulnerability to the stressors that accompany aging. Additionally, our findings underscore the role of systemic inflammation in frailty, with CRP emerging as a significant independent predictor. This aligns with prior studies highlighting CRP as a biomarker of chronic inflammation associated with frailty in aging populations [26]. The pro-inflammatory state reflected by the elevated CRP levels, often termed ‘inflammaging,’ exacerbated the physiological decline linked to frailty [26,27]. These results, together with the established role of age, provide further evidence of the interplay between inflammation and aging in the pathogenesis of frailty in heart failure.

A systematic review by Feng et al. (2017) analyzed the factors associated with frailty in community-dwelling individuals aged 60 and older across 23 longitudinal studies. The findings highlighted significant correlations with frailty across several domains: sociodemographic factors (noted in all seven studies reviewed), which encompassed older age, ethnic background, neighborhood characteristics, and access to private insurance or Medicare; physical factors (identified in five out of six studies), including obesity and the activities of daily living (ADL) status; biological factors (reported in five out of seven studies), such as serum uric acid levels; lifestyle factors (found in 11 out of 13 studies), which included a higher Diet Quality Index International score, greater fruit and vegetable intake, and higher dietary resveratrol exposure; and psychological factors (observed in seven out of eight studies), particularly depressive symptoms [28]. These findings underscore the multifactorial nature of frailty, highlighting the importance of a broad range of influences, from dietary habits to socioeconomic status. In the context of our findings, the systematic review by Feng et al. (2017) provides a robust framework to understand the multifaceted nature of frailty in elderly populations, particularly in those suffering from HF. Our study further emphasizes the complexity of frailty by identifying the age, NYHA class, length of the hospital stay, and psychological factors such as anxiety and depression scores as significant predictors of frailty among patients with HF. These results align closely with Feng et al.’s findings, which demonstrate the impact of sociodemographic, physical, and psychological factors on frailty [28]. In our study, diabetes mellitus was the only comorbid condition that showed significant differences between frail and non-frail patients. Interestingly, its prevalence was lower in the frail group, a finding that diverges from the broader literature associating diabetes with increased frailty risk [29,30]. This discrepancy may be attributed to differences in patient selection criteria or underlying characteristics, highlighting the need for further research into the interplay between diabetes and frailty in heart failure populations. Additionally, pharmacological strategies tailored for frail patients with heart failure may offer significant benefits. Emerging therapies such as sodium-glucose co-transporter-2 inhibitors (SGLT2is) and angiotensin receptor-neprilysin inhibitors (ARNIs) have demonstrated efficacy in improving outcomes for heart failure patients, including those with comorbidities such as diabetes mellitus and chronic kidney disease. Recent studies, including a network meta-analysis by Lavalle et al., underscore the role of SGLT2is like dapagliflozin and empagliflozin in reducing hospitalization rates and cardiovascular mortality in frail HF populations [31]. These drugs, alongside ARNIs, could represent key components of a comprehensive treatment approach for frail patients, addressing both cardiac and systemic vulnerabilities in this high-risk group.

In our study, we observed a distribution of HF types—HFrEF, HFmrEF, and HFpEF—among an older patient population, which is in line with the current epidemiological trends reported in the literature. The prevalence of HFpEF in our cohort mirrors findings from broader community-based studies, emphasizing its increasing recognition in older adults [32]. HFpEF is particularly associated with age-related comorbidities such as hypertension, obesity, and diabetes, which were also prevalent in our study group [32,33]. This complex interplay of HF types and associated comorbidities in our older patients highlights the need for nuanced management strategies that address both the cardiac and systemic health challenges they face [32].

The NYHA class also emerged as a significant predictor, which is insightful for clinical practice. As HF progresses, marked by higher NYHA classes, the physical limitations increase, possibly accelerating the onset of frailty. This finding underscores the importance of early intervention in HF management to potentially delay the progression of both HF and frailty [34].

The length of the hospital stay is another significant predictor, indicating the severity of the episodes leading to hospitalization. Longer hospital stays may reflect more severe acute exacerbations of HF or complications that could exacerbate frailty [35]. This association suggests that reducing hospitalization through effective management strategies could potentially impact the progression of frailty. In our study, we explored the connection between frailty and the duration of hospital stays in patients with HF, considering the broader context of frailty’s impact on health outcomes. Our analysis reveals that the length of hospitalization is significantly associated with frailty, even after adjusting for other well-established prognostic factors. This indicates that frailty, as a distinct clinical entity, adds a layer of complexity to the management of patients with HF and their healthcare utilization.

Our analysis showed that the patients identified with higher frailty scores were consistently associated with longer hospitalizations. Unlike the studies that focus solely on individual frailty components, our approach, which integrated multiple aspects of frailty into a comprehensive index, underscores the multifaceted nature of frailty in predicting hospitalization needs. This correlation between a comprehensive frailty assessment and prolonged hospital stays in patients with HF suggests that interventions targeting frailty may offer a pathway to optimize hospital resource use and improve patient outcomes. Recognizing the role of frailty in this context could lead to more personalized and effective management strategies for patients with HF, potentially mitigating the risk of prolonged hospitalizations and enhancing overall treatment efficacy [36]. In parallel with our findings on the impact of frailty on hospitalization duration in patients with HF, the study by Leong et al. further elucidates the intricate relationship between frailty and hospitalization [37]. Leong and colleagues found that the association between frailty and HF hospitalization remains robust and independent of other known prognostic factors, such as age, comorbidity burden, and baseline heart function. Their use of a comprehensive frailty index—which integrates various frailty components—demonstrated that a holistic assessment of frailty could identify patients at a higher risk of adverse hospitalization outcomes more effectively than individual frailty components or smaller combinations thereof [37].

Our study supports and extends these findings by showing that longer hospital stays are not merely a function of the number of comorbidities or severity of HF but are significantly influenced by the broader concept of frailty. Like Leong et al., we observed that frailty plays a critical role in determining hospitalization outcomes [37].

The approach taken by Leong et al. in employing a full frailty index to gauge hospitalization risks underscores the utility of comprehensive frailty assessments in clinical practice. This aligns with our suggestion that the early identification and management of frailty in patients with HF can lead to more personalized and effective care strategies, potentially reducing hospital stay durations and improving overall health outcomes. Incorporating a similar frailty index into routine clinical assessments could offer a predictive advantage, helping healthcare providers better anticipate and mitigate the risks associated with hospitalizations in this vulnerable patient population. As such, our study, alongside the insights from Leong et al., advocates for a shift towards integrating frailty evaluations in the standard care protocols for patients with HF, aiming to improve prognostic accuracy and patient-centered care outcomes [37]. 

Furthermore, psychological factors such as anxiety and depression were significant predictors of frailty. These findings align with the broader literature indicating that mental health significantly impacts physical health, especially in chronic conditions like HF. The relatively low depression scores observed in our study differ from those reported by Uchmanowicz et al. [38] and Warraich et al. [39]. Warraich et al. reported that 38% of the patients hospitalized for heart failure presented with significant depressive symptoms measured by GDS, often unrecognized in clinical records [39]. Similarly, Uchmanowicz et al. found that 28% of the elderly patients with atrial fibrillation exhibited depressive symptoms on the HADS, highlighting the strong correlation between depression and frailty [38]. These discrepancies may be attributed to the differences in study populations and methodologies. Our study specifically excluded patients with a prior diagnosis of depression and conducted assessments during clinical stabilization, which could contribute to lower observed HADS scores. Despite these differences, our findings align with the broader literature underscoring the interplay between depression and frailty. Anxiety and depression may contribute to a reduced capacity to engage in physical activity, poorer self-care, and less social interaction, all of which can hasten the decline into frailty. In our findings, anxiety emerged as a noteworthy independent predictor affecting the health outcomes of patients with HF. This aligns with existing research indicating that anxiety not only worsens patients’ functional status but also significantly detracts from their health-related quality of life (HRQoL) and leads to more frequent rehospitalizations. Depression and anxiety predict the decline in physical health functioning in patients with HF [10,33,40]. The evidence from our study emphasizes the need for comprehensive management strategies that include robust psychological support to mitigate these impacts. Addressing anxiety and other psychological factors in patients with HF is crucial, not only for improving their quality of life but also for reducing the incidence of hospital readmissions, thereby alleviating the overall strain on healthcare resources.

These results highlight the need for a comprehensive approach to managing HF that includes not only addressing the physical aspects of the disease but also focusing on mental health and strategies to reduce hospitalization time. Addressing these factors might not only improve the quality of life and functional status of these patients but could also delay or mitigate the onset of frailty. Thus, interventions should be tailored to address both the physical and psychological needs of patients, ensuring a holistic approach to care that may help maintain their independence for longer periods.

Furthermore, the results of the presented study emphasize the complex interplay between clinical, psychological, and demographic factors in determining frailty among HF patients. Further studies are required to explore these associations to improve patient care and outcomes.

### 4.1. Implications for Clinical Practice

- Management of Cognitive and Mood Disorders

Effective management strategies include the integration of cognitive and psychological evaluations into standard cardiovascular care practices. Early identification of mood disorders and cognitive impairments can facilitate timely interventions, potentially slowing the progression of HF and improving patient outcomes.

- Integrative Care Approaches

The findings advocate for integrative care approaches that address the psychological, cognitive, and physical aspects of health. This could involve multidisciplinary teams that coordinate to provide comprehensive care tailored to the needs of cardiovascular patients, particularly those at a high risk of frailty and cognitive decline.

### 4.2. Study Limitations

This study acknowledges several limitations. First, potential biases in self-reported data may have influenced the results, as patients may underreport or overreport their symptoms and health status. Second, the cross-sectional nature of some data sets limits the ability to draw causal inferences about the relationships observed. Third, the study sample was limited to patients from a specific geographic region and hospital, which may affect the generalizability of the findings to other populations. Additionally, the inclusion criteria focused on patients aged 60 years and older with specific NYHA classes and cognitive function, potentially excluding younger patients or those with more severe cognitive impairments. Another limitation concerns the relatively low depression scores observed in the HADS, as presented in Table 3. These results may have been influenced by the exclusion of patients with diagnosed and treated depression, potentially limiting the generalizability of the findings to broader populations with heart failure. The next limitation of this study is that the potential influence of medication use or other interventions during hospitalization on cognitive and frailty assessments was not specifically controlled. Finally, this study did not specifically evaluate multicollinearity among the variables in the regression model, which could potentially affect the stability of the results and their interpretation. Future studies should address this issue to enhance the robustness of findings.

## 5. Conclusions

Age, CRP levels, and anxiety act as independent predictors of frailty in patients with heart failure. Other factors, such as depression, cognitive dysfunction, and the length of the hospital stay, showed significant differences between the frail and non-frail patients in the group comparisons, but were not independent predictors of frailty. These results highlight the complexity of frailty in older adults with heart failure, where psychological and clinical factors play significant roles. Future studies are needed to confirm these findings and explore the causal pathways linking these variables.

## Figures and Tables

**Table 1:** Demographic characteristics of the sample, overall and by level of frailty. Descriptive data were presented as mean ± SD (±standard deviation) or number of observations (percent).

Variables	All Patients (*n* = 250)	Frail (*n* = 151)	Non-Frail (*n* = 99)	*p*-Value (*t*-Student or Chi^2^ Test)
Age (years)	72.32 ± 6.73	73.58 ± 6.80	70.39 ± 6.16	0.0002 *
Gender:				0.328
Female	77 (30.80)	50 (33.11)	27 (27.27)	
Male	173 (69.20)	101 (66.89)	72 (72.73)	
Marital status:				0.297
In a relationship	165 (65.60)	96 (63.58)	69 (69.70)	
Single	85 (34.00)	55 (36.42)	30 (30.30)	
Residence:				0.642
Urban	188 (75.20)	112 (74.17)	76 (76.77)	
Rural	62 (24.80)	39 (25.83)	23 (23.23)	
Work status:				0.285
Professionally active	31 (12.40)	16 (10.60)	15 (15.15)	
Pensioner	219 (87.60)	135 (89.40)	84 (84.85)	
Education years	12.25 ± 3.67	12.07 ± 3.77	12.53 ± 3.51	0.331
BMI (kg/m^2^)	29.00 ± 5.71	28.78 ± 5.93	29.34 ± 5.38	0.448
Central obesity (waist circumference > 94 cm for man, >80 cm for woman):				0.592
No	44 (17.60)	25 (16.56)	19 (19.19)	
Yes	206 (82.40)	126 (83.44)	80 (80.81)	
Smoking:				0.477
No	211 (84.40)	131 (86.75)	80 (80.80)	
Yes	39 (15.60)	20 (13.25)	19 (19.19)	

*: statistically significant; BMI: Body Mass Index.

**Table 2:** Clinical characteristics, comorbidities, laboratory parameters, and medications in the patients with or without frailty syndrome. Descriptive data were presented as the mean ± SD (±standard deviation), median [Q1–Q3], or number of observations (percent).

Variables	All Patients (*n* = 250)	Frail (*n* = 151)	Non-Frail (*n* = 99)	*p*-Value (*t*-Student, Mann–Whitney ^(1)^ or Chi^2^ Test)
NYHA class:				0.001 *
II	109 (43.60)	55 (36.42)	54 (54.55)	
III	99 (39.60)	61 (40.40)	38 (38.38)	
IV	42 (16.80)	35 (23.18)	7 (7.07)	
LVEF (%)	44.00	44.00	45.00	0.415 ^(1)^
	[33.00–56.00]	[32.00–55.00]	[33.00–60.00]	
HF Phenotype:				0.822
HFrEF	105 (42.00)	65 (43.05)	40 (40.40)	
HFmrEF	46 (18.40)	26 (17.22)	20 (20.20)	
HFpEF	99 (39.60)	60 (39.74)	39 (39.39)	
Length of hospital stay (days)	5.00	5.00	5.00	0.003 *^(1)^
	[5.00–6.00]	[5.00–7.00]	[5.00–5.00]	
Comorbidities				
Hypertension	222 (88.80)	133 (88.08)	89 (89.90)	0.655
CAD	159 (63.60)	89 (58.94)	70 (70.71)	0.058
MI	99 (39.60)	61 (40.40)	38 (38.38)	0.750
Diabetes mellitus	113 (45.20)	60 (39.74)	53 (53.54)	0.032 *
Bronchial asthma	45 (18.00)	30 (19.87)	15 (15.15)	0.447
Kidney diseases	101 (40.40)	65 (43.04)	36 (36.36)	0.439
Liver diseases	25 (10.00)	16 (10.60)	9 (9.09)	0.698
CVA	33 (13.20)	18 (11.92)	15 (15.15)	0.460
CTD	56 (22.40)	39 (25.83)	17 (17.17)	0.108
PUD	31 (12.40)	23 (15.23)	8 (8.08)	0.093
Cancer	43 (17.2)	31 (20.53)	12 (12.12)	0.084
CVDs in family	69 (27.60)	38 (25.17)	31 (31.31)	0.287
Laboratory parameters:				
Hemoglobin (g/dL)	13.63 ± 1.82	13.41 ± 1.78	13.97 ± 1.84	0.016 *
Albumin (g/dL)	3.70	3.70	3.60	0.816 ^(1)^
	[3.40–4.00]	[3.40–4.00]	[3.50–3.90]	
CRP (mg/L)	3.49	4.68	2.54	<0.0001 *^(1)^
	[1.57–7.34]	[2.21–8.97]	[1.07–4.77]	
NT-proBNP (pg/mL)	1653.80	2558.80	1102.60	<0.0001 *^(1)^
	[750.10–4928.50]	[943.90–5937.20]	[605.40–3001.50]	
eGFR (mL/min/1.73 m^2^)	66.50	62.00	71.00	0.024 *^(1)^
	[51.00–84.00]	[47.00–82.00]	[54.00–89.00]	
Creatinine (mg/dL)	1.30 ± 0.99	1.34 ± 0.98	1.25 ± 1.01	0.492
BUN (mg/dL)	45.00	44.50	45.50	0.361 ^(1)^
	[36.00–63.00]	[37.00–68.00]	[35.00–58.00]	
Medication at discharge:				
ACE inhibitors/ARB	246 (98.40)	149 (98.67)	97 (97.97)	0.406
Calcium channel blockers	61 (24.40)	38 (25.16)	23 (23.23)	0.308
Alpha-blockers	20 (8.00)	13 (8.61)	7 (7.07)	0.639
Beta-blockers	249 (99.60)	150 (99.33)	99 (100.00)	0.465
MRA	191 (76.40)	119 (78.80)	72 (72.72)	0.026 *
Diuretics	228 (91.20)	137 (90.72)	91 (91.91)	0.313
Statins	221 (88.40)	134 (88.74)	87 (87.87)	0.570
Anticoagulant agents	163 (65.20)	104 (68.87)	59 (59.59)	0.666
Antiplatelet agents	111 (44.40)	62 (41.05)	49 (49.49)	0.514

*: statistically significant; ^(1)^: Mann–Whitney test; ACE: angiotensin-converting enzyme; ARB: angiotensin receptor blockers; BUN: blood urea nitrogen; CAD: coronary artery disease; CRP: C-reactive protein; CTD: connective tissue diseases; CVA: cerebrovascular accident; CVDs: cardiovascular diseases; eGFR: estimated glomerular filtration rate; HFrEF: heart failure with reduced ejection fraction; HFmrEF: heart failure with mildly reduced ejection fraction; HFpEF: heart failure with preserved ejection fraction; LVEF: left ventricular ejection fraction; MI: myocardial infarction; MRAs: antagonists of mineralocorticoid receptors; NT-proBNP: N-terminal pro-B-type natriuretic peptide; NYHA: New York Heart Association; PUD: peptic ulcer disease.

**Table 3:** Differences in the MMSE, MoCA, HADS, and PHQ-9 scales in patients with HF were divided into two groups according to the FS presence. Descriptive data were presented as the number of observations.

	All Patients (*n* = 250)	Frail (*n* = 151)	Non-Frail (*n* = 99)	*p*-Value (Chi^2^ Test)
MMSE:				0.008 *
No CI (30–27)	187 (74.80)	105 (69.54)	82 (82.83)	
CI without dementia (26–24)	52 (20.80)	35 (23.18)	17 (17.17)	
Mild dementia (23–19)	11 (4.40)	11 (7.28)	0 (0.00)	
Medium-grade dementia (18–11)	0 (0.00)	0 (0.00)		
High-grade dementia (10–0)	0 (0.00)	0 (0.00)		
MoCA				0.018 *
No CI (30–26)	126 (50.40)	67 (44.37)	59 (59.60)	
CI (<25)	124 (49.60)	84 (55.63)	40 (40.40)	
HADS Anxiety				0.002 *
No disorder (0–7)	224 (89.60)	127 (84.11)	97 (97.98)	
Borderline result (8–10)	12 (4.80)	11 (7.28)	1 (1.01)	
Marked disorder (11–21)	14 (5.60)	13 (8.61)	1 (1.01)	
HADS Depression				0.021 *
No disorder (0–7)	220 (88.00)	126 (83.44)	94 (94.95)	
Borderline result (8–10)	21 (8.40)	17 (11.26)	4 (4.04)	
Marked disorder (11–21)	9 (3.60)	8 (5.30)	1 (1.01)	
PHQ-9				<0.001 *
No disorder (0–4)	115 (46.00)	53 (35.10)	62 (62.63)	
Mild (5–9)	91 (36.40)	61 (40.40)	30 (30.30)	
Moderate (10–14)	25 (10.00)	20 (13.25)	5 (5.05)	
Moderately severe (15–19)	14 (5.60)	13 (8.61)	1 (1.01)	
Severe (20–27)	5 (2.00)	4 (2.65)	1 (1.01)	

*: statistically significant; CI: cognitive impairment; MMSE: Mini-Mental State Examination; MoCA: Montreal Cognitive Assessment; HADS: Hospital Anxiety and Depression Scale; PHQ-9: Patient Health Questionnaire-9.

**Table 4:** Univariable and multivariable logistic regression analysis for predictors of frailty syndrome presence in patients with HF.

Variable	Univariable Analysis		Multivariable Analysis	
	OR (95% CI)	*p*-Value	OR (95% CI)	*p*-Value
Age (for >70.5 years old)	2.62	0.0003 *	2.85	0.001 *
	(1.55–4.45)		(1.50–5.40)	
NYHA (for IV)	3.96	0.002 *	1.87	0.206
	(1.67–9.37)		(0.70–4.98)	
Length of hospital stay (for >6 days)	2.88	0.004 *	2.16	0.082
	(1.39–5.96)		(0.90–5.22)	
Diabetes mellitus	1.74	0.032 *	1.61	0.127
	(1.04–2.92)		(0.86–3.01)	
Hemoglobin (for <13 g/dL)	1.84	0.034 *	1.14	0.699
	(1.04–3.25)		(0.58–2.24)	
CRP (for >5 mg/L)	2.85	<0.001 *	2.00	0.045 *
	(1.61–5.04)		(1.00–3.98)	
eGFR (for <60 mL/min/1.73 m^2^)	1.71	0.046 *	0.94	0.864
	(1.00–2.91)		(0.49–1.79)	
HADS Anxiety (for >7 points)	9.16	0.003 *	8.71	0.008 *
	(2.09–40.01)		(1.70–44.38)	
HADS Depression (for >7 points)	3.73	0.009 *	0.98	0.977
	(1.37–10.15)		(0.28–3.44)	
PHQ-9 (for >4 points)	3.09	<0.0001 *	2.47	0.004 *
	(1.82–5.26)		(1.31–4.63)	
MoCA (for <26 points)	1.84	0.019 *	1.34	0.364
	(1.10–3.09)		(0.70–2.53)	
MMSE (for <26 points)	1.90	0.091	1.36	0.508
	(0.89–4.02)		(0.54–3.43)	

*: statistically significant; CRP: C-reactive protein; eGFR: estimated glomerular filtration rate; HADS: Hospital Anxiety and Depression Scale; MMSE: Mini-Mental State Examination; MoCA: Montreal Cognitive Assessment; NYHA: New York Heart Association; PHQ-9: Patient Health Questionnaire-9.

## Data Availability

The data can be obtained by contacting the corresponding author.
